# Evaluating the solution from *MrBUMP* and *BALBES*

**DOI:** 10.1107/S0907444911007530

**Published:** 2011-03-18

**Authors:** Ronan M. Keegan, Fei Long, Vincent J. Fazio, Martyn D. Winn, Garib N. Murshudov, Alexei A. Vagin

**Affiliations:** a STFC Rutherford Appleton Laboratory, Chilton OX11 0FA, England; b York Structural Biology Laboratory, Chemistry Department, University of York, Heslington, York, England; c CSIRO Molecular and Health Technologies, Parkville, Australia; d STFC Daresbury Laboratory, Daresbury, Warrington WA4 4AD, England

**Keywords:** *MrBUMP*, *BALBES*, molecular replacement

## Abstract

Molecular replacement is one of the key methods used to solve the problem of determining the phases of structure factors in protein structure solution from X-ray image diffraction data. Its success rate has been steadily improving with the development of improved software methods and the increasing number of structures available in the PDB for use as search models. Despite this, in cases where there is low sequence identity between the target-structure sequence and that of its set of possible homologues it can be a difficult and time-consuming chore to isolate and prepare the best search model for molecular replacement. *MrBUMP* and *BALBES* are two recent developments from *CCP*4 that have been designed to automate and speed up the process of determining and preparing the best search models and putting them through molecular replacement. Their intention is to provide the user with a broad set of results using many search models and to highlight the best of these for further processing. An overview of both programs is presented along with a description of how best to use them, citing case studies and the results of large-scale testing of the software.

## Abstract 2

The automated pipelines for molecular replacement *MrBUMP* and *BALBES* are reviewed, with an emphasis on understanding their output. Conclusions are drawn from their performance in extensive trials.

## 1. Introduction

Deriving the structure of macromolecules from X-ray experiments presents many challenges to the protein crystallo­grapher. To solve a structure, two key pieces of information are required: the amplitudes of the structure factors, which are derived from the intensity information of the X-ray diffraction images, and the phases of the structure factors, which must be elicited through other means. Where a related structure or homologous structure for the target structure already exists, usually the easiest and most convenient method to use is known as molecular replacement (MR). Recent years have seen rapid advances in MR techniques that have helped to improve the success rate of the method. In spite of this, in limiting cases (*e.g.* when the search models are highly mobile with many domains and/or subunits or the sequence identity between the search and target sequences is very small, *i.e.* ≤25%) many search models, including domain-based and multimer-based models, may need to be processed before a solution is found. Performing this can be very time-consuming and tedious. Two developments by CCP4 (Collaborative Computational Project, Number 4, 1994 ▶; Winn *et al.*, 2011 ▶) seek to automate the process of molecular replacement. *BALBES* (Long *et al.*, 2008 ▶) automates the process of identifying the best possible search model from its own customized version of the PDB database, prepares it and puts it through molecular replacement. *MrBUMP* (Keegan & Winn, 2007 ▶, 2008 ▶) takes a broader approach to the problem: it identifies as many reasonable search models from the PDB as possible, prepares the best of them in a number of different ways and puts them through molecular replacement. Here, we will give a brief description of both of these programs, how to use them and what to look for in their output.

## 2. *BALBES*

*BALBES* has been developed by the software team at the York Structural Biology Laboratory (YSBL) at York University in the UK since 2007.

### 2.1. Components of *BALBES*

*BALBES* consists of three components: (i) a database of protein structures specifically designed for MR, (ii) a pipeline manager written in Python and (iii) a group of executable programs such as *MOLREP* (Lebedev *et al.*, 2008 ▶; Vagin & Teplyakov, 2010 ▶) and *REFMAC* (Murshudov *et al.*, 1997 ▶, 2011 ▶) used as the engine. The following sections describe (i) and (ii). A more comprehensive description of the program can be found in Long *et al.* (2008 ▶).

#### 2.1.1. The *BALBES* database: selection of entries

All protein entries from the PDB with a length greater than 15 amino-acid residues that had been solved using macromolecular crystallography and had been refined against data to better than 3.5 Å resolution were selected to build the current database. The basic entries in the database are macromolecular subunits. If two subunits had a sequence identity of greater than 80% and a root-mean-square deviation (r.m.s.d.) between corresponding C^α^ atoms of less than 1 Å then the one that had been refined against higher resolution data was retained. This approach, while substantially reducing the number of entries kept in the database, retained the conformational variability of the molecules. For example, if there were two copies of a molecule and there was a domain motion during binding of a substrate then both representatives were kept in the database, even if the sequence identity was 100%. Currently, there are about 28 000 unique chain entries in the database that are selected from among about 69 000 protein entries in the PDB.

The resulting chains are organized in a hierarchical manner using a sequence alignment that was corrected after a domain alignment. Thus, the alignment contains information about sequence as well as three-dimensional similarities. All of the chains have been manually checked and domains are defined according to their spatial compactness. Domains are also organized in a hierarchical manner using a three-dimensional alignment. The multimeric organization of each chain is also stored in the form of operators. Information about multimers was taken from *PISA* (Krissinel & Henrick, 2005 ▶). The current version of the database contains around 30 000 domains and 13 500 multimer specifications. The database is updated every month. During the update, domains are first defined automatically using the domain database and then corrected manually if needed.

#### 2.1.2. The *BALBES* manager

The *BALBES* manager is written in Python; it controls the flow of information and selects the protocols used to process it. The manager takes in reflection data and sequence files. Firstly, the sequences of potentially similar models are extracted from the database. For each model, information about its domain as well as its multimeric organization are also extracted. For each domain, an ensemble is generated using the domain database. A full description of the ensemble generation is outside the scope of this paper and will be published elsewhere. These ensembles are the search models. Using the sequence alignment, the model is corrected using the options available in *MOLREP* (Lebedev *et al.*, 2008 ▶).

### 2.2. *BALBES* usage

There are three ways to use *BALBES*.

(i) A simple command line; the most typical one is balbes -­o outpath -f structural_factor_data.mtz -s sequence_data.seq. *BALBES* tries to solve the structure using the input structure-factor amplitudes (MTZ or CIF format) and sequence (*FASTA* format) information. Users can also define their own library of PDB files in the command line. *BALBES* can also be used to find similar structures in the database. These methods are shown on the York software website (http://www.ysbl.york.ac.uk/YSBLPrograms/index.jsp).
(ii) The *ccp*4*i* interface (Potterton *et al.*, 2004 ▶; Winn *et al.*, 2011 ▶), in which a user needs to enter the structure-factor amplitudes and sequence files. Once a job has been submitted, no user intervention is needed.
(iii) *Via* the York Structural Biology Laboratory web server available at http://www.ysbl.york.ac.uk/YSBLPrograms/index.jsp.

The best solution found by *BALBES* can be sent directly to the *ARP*/*wARP* (Langer *et al.*, 2008 ▶) web service for automatic model building. The web server also offers users options to carry out space-group checking and runs different space-group candidates in parallel on the cluster of computers in York. When all of the space groups have been checked, the program takes the space group that gives the solution with the highest score (see below).

### 2.3. How does *BALBES* work?

#### 2.3.1. Template search models for molecular replacement

At the beginning of a structural solution process, *BALBES* searches the database using an algorithm that uses pairwise dynamic sequence alignment and the hierarchical clusters of chains and domains in the database and provides a set of template models for MR. The details of the algorithm used are described by Long *et al.* (2008 ▶). These models are grouped according to their association with the target sequences, the original PDB files that they come from and their multimeric and domain organizations. *BALBES* indexes these models using IDs such as ‘assembly’, ‘sequence’, ‘structure (PDB)’ and ‘model No.’.

**Figure 1:**
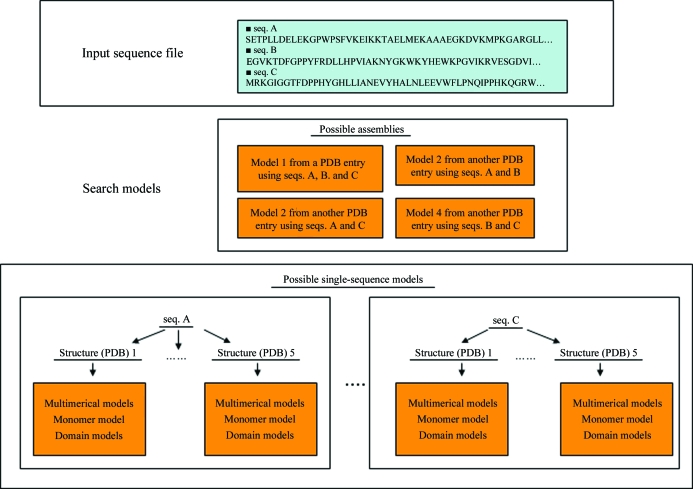
The types and hierarchy of the search models presented in *BALBES* jobs.

Fig. 1 ▶ shows an example of the hierarchical order in which these models are organized in *BALBES* for a case where three target sequences have been provided. *BALBES* tries to search the database for the assembly models using all three as well as any combination of two sequences. Once such models are found, *BALBES* outputs details of these models, as shown in Table 1 ▶(*a*). It can be seen that the assembly model given in Table 1 ▶(*a*) is obtained by using sequences 1, 2 and 3. The model comes from PDB file 2a74 and contains 1109 residues. The overall sequence similarity is 54%. It is expected to have four copies of such an assembly molecule in the unit cell.

In addition to these assemblies, *BALBES* also tries to find models associated with each sequence. As shown in Fig. 1 ▶, models for up to five different structures (PDB files) are given for each sequence. For each structure (PDB file), models of multimers, monomers and domains are presented. An example of models from one structure (PDB file) using one sequence is shown in Table 1 ▶(*b*). It can be seen that *BALBES* finds one dimer, one monomer and two domains, all of which have a sequence similarity of 1.0, from PDB file 1n9w. If ‘(ENS)’ is shown in the table it means that the chains or domains of that structure are used to generate ensembles. The details of ensemble generation will be published elsewhere. *BALBES* uses the ensembles first and if a solution is not found it then switches to single-chain models.

Another set of search models provided by *BALBES* is shown in Table 1 ▶(*c*). Unlike the models in Table 1 ▶(*b*), these models are all domains from different structures (PDB files).

Finally, *BALBES* uses the search models generated on the fly during the later stages of the structure-solution process. For example, when there are several sequences and no assembly models of multiple chains are found or the assembly models found did not give good solutions, *BALBES* works on all of the search models generated using the single sequences. It keeps the best solution for each sequence. It then fixes the best solution of one sequence and uses the best solution of another sequence as the search model in turn until all of the best solutions are used. In this case, the search models are actually the solution generated in the previous step. Those solution structures could be a combination of multiple domains which are different from the original models. A detailed example is shown in §[Sec sec2.3.2]2.3.2.

#### 2.3.2. Protocols and criteria for structure solution

**Figure 2:**
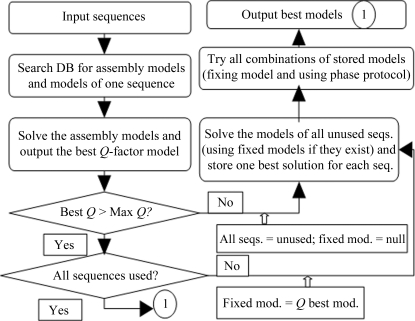
A flow chart of the structural solution process in *BALBES*.

The protocols for *BALBES* to find a structure solution are briefly described in the flow chart shown in Fig. 2 ▶. Basically, if multiple sequences are provided *BALBES* tries the assembly models associated with multiple sequences first. If this is not successful it then tries all models associated with each chain and stores the best model for each sequence. It then combines all of the best models to give the final solution.

As we can see from the flow chart (Fig. 2 ▶), a quality factor *Q* is used to define the direction of the solution process and the final decision for the best solution. The *Q* factor is calculated after refinement using the following empirical formula, 
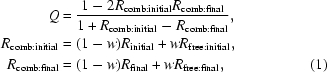
where *w* = 0.75 and *R* and *R*_free_ are the *R* and free *R* factors. Once the *Q* is calculated, the solutions are assigned a probability using the formula
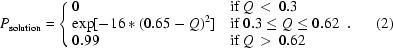
In practice, this formulation seems to work sufficiently well. If *Q* > 0.75 *BALBES* has found a solution that is definitely correct. If *Q* < 0.25 the solution found is very unlikely to be correct. When *Q* falls between 0.25 and 0.75 the probability given by *P*_solution_ shows how likely the solution is to be correct. However, the direct link between a partial solution and *Q* has not been established. Furthermore, it does not take into account crystal-growth peculiarities such as twinning and pseudo-translation and therefore the scores may be mis­leading in these cases. A better criterion could be established *via* theoretical modelling and statistical data-analysis tech­niques based on the data from our routine test cases.

Table 2 ▶ shows an example of the result at the end of structural solution, in which the *Q* factor and its associated probability are presented. Table 2 ▶ also shows that the structure was solved using assembly model 1 (model index as1m1).

### 2.4. Tests on *BALBES* and a case study

Every half a month we take the newly released data from the PDB. Before putting the relevant entries into the database, we use the structure-factor amplitudes and sequences to test *BALBES* with the existing database. Solutions found by *BALBES* are compared with the deposited PDB file. We have continually carried out these tests for more than three years. Our test results show that *BALBES* is able to solve about 80% of deposited structures automatically. Table 3 ▶ shows the results of our last six tests during 2010 (the tests scheduled to start on 15 July and 15 September were cancelled because of computing problems). It should be pointed out that structures deposited in the PDB represent only a (solvable) fraction of all X-ray crystallography data. Therefore, the statistics in Table 3 ▶ contain a certain degree of bias.

**Figure 3:**
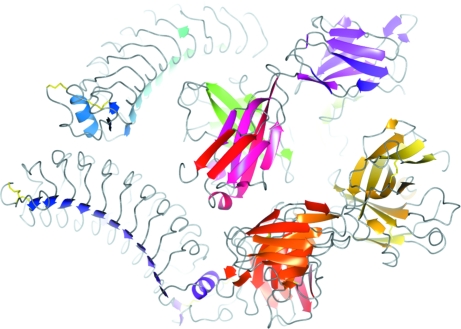
An example shows how *BALBES* combines models from different PDB files to obtain a complex solution.

Fig. 3 shows an example of a complex structure solution from the test cases. The input file contains three sequences of 219, 219 and 283 residues, respectively. *BALBES* finds three assemblies, with each containing two sequences. However, these models do not result in high-quality solutions. *BALBES* then works on the models generated from each sequence. 35 models have been tried, which come from 15 different PDB files with similarity ranging from 0.26 to 1.0. The best solutions are generated by the monomer models from 1nld, 1ozn and 1nlb. *BALBES* then takes these three best solutions and tries using one solution as the starting fixed model and the others in turn as the search models to perform MR and refinement. The final solution is indexed as sq2st2m1_sq3st1m1_sq1st4m1, which means it is obtained by first fixing the best solution from sequence 2 and using that from sequence 3 as the search model and then fixing the new solution from that and using the best solution from sequence 1 as the search model. Fig. 3 ▶ shows the final resultant structure and Table 4 ▶ gives a summary of the final results.

The algorithms in *BALBES* are also able to handle models with low sequence similarity in some cases. Table 5 ▶ shows the success rates for low sequence similarity (<0.30) in the last six rounds of tests.

The time spent to solve a structure using *BALBES* varies significantly from case to case depending on many factors, *e.g.* the number and sizes of the search models found. For a case of moderate difficulty, such as the case shown in Table 1 ▶, *BALBES* completed all of the model searching, MR and refinement on all search models in about 2 h on a laptop with an Intel Core 2 Duo 2.0 GHz CPU.

## 3. *MrBUMP*

*MrBUMP* (**M**olecular **r**eplacement with **BU**lk **M**odel **P**reparation) was originally developed as part of the e-HTPX project for high-throughput protein crystallography in the UK (Allan *et al.*, 2005 ▶). When this project matured, the *MrBUMP* software was taken over by the CCP4 group for its long-term support and development. It is designed to perform MR on target data using a large set of search models. In straight­forward cases, where the sequence identity between the target and its set of close homologues is high (>60%), *MrBUMP* should identify pertinent search models, prepare them and produce a molecular-replacement solution that in its refinement clearly shows the potential for a solution. In less clear-cut cases the exhaustive nature of *MrBUMP* provides a means for a user to explore a large parameter space. It has been found that this approach has successfully produced a solution in several cases (Karbat *et al.*, 2007 ▶; Wang *et al.*, 2008 ▶; for details, see Keegan & Winn, 2007 ▶) where a solution was not obvious. Here, we will only give a brief description of how the program works. For a more comprehensive description of the program, see the original publications on *MrBUMP* (Keegan & Winn, 2007 ▶, 2008 ▶).

### 3.1. Using *MrBUMP*

*MrBUMP* can be run in the typical *CCP*4 style, either from the command line or through its own *ccp*4*i* interface. When run from the command line, input and output files can be specified through command-line arguments with program-control parameters specified through keyword arguments. The basic command line is as follows: mrbump HKLIN input.mtz HKLOUT output.mtz SEQIN sequence.seq XYZOUT output.pdb. Documentation on all of the possible keywords is available in the mrbump_doc.html file included in the *CCP*4 suite or *via* the program-documentation area on the CCP4 website (http://www.ccp4.ac.uk). The *ccp*4*i* interface requires the specification of input and output files and provides access to many of the underlying keywords with sensible defaults for each of them.

Once running, the program follows a simple pipeline. The target data are processed for necessary information and a *FASTA* search (Pearson & Lipman, 1988 ▶) using the target sequence is performed to identify possible homologues, with the best of these selected for further processing. The corresponding PDB files for each of the best *FASTA* hits, referred to as template search models, are retrieved and prepared for molecular replacement using a number of possible methods. These methods include the use of the programs *CHAINSAW* (Stein, 2008 ▶) and *MOLREP* (Vagin & Teplyakov, 1997 ▶) to prune the nonconserved side chains of the template search models according to the sequence alignment of the target with the template. These so-called ‘mixed models’ often prove to be the most likely to succeed in MR (Schwarzenbacher *et al.*, 2004 ▶). In addition, domains within the set of *FASTA* results are identified using *SCOP* (Murzin *et al.*, 1995 ▶; Lo Conte *et al.*, 2002 ▶) and multimeric forms of the *FASTA* results are identified using the *PQS* server at the EBI (http://www.ebi.ac.uk). These are all added to the list of template search models. They may provide solutions in molecular replacement where the parent chain fails. *Phaser* (McCoy *et al.*, 2005 ▶), which is one of the programs available to *MrBUMP* for performing the molecular-replacement processing, has the option to take in ensembles of superpositioned models. *MrBUMP* will create an ensemble model from the best of the template search models. In addition to the set of search models automatically generated by the program, the user has the option to input their own PDB files as well as providing the PDB codes of structures that they may know to be suitable.

At this point in the pipeline we have a set of search models ready for passing into MR. The list of search models is ranked according to a scoring function based on their sequence alignment with the target sequence and the completeness of the alignment (Keegan & Winn, 2007 ▶). This ranking determines the order in which the models are processed in MR. *Phaser* or *MOLREP* or both can be used to perform the MR procedure. For each search model, if the processing is successful the resulting positioned model is subjected to 30 cycles of restrained refinement in *REFMAC* (Murshudov *et al.*, 1997 ▶, 2011 ▶) to ascertain its potential as a solution.

#### 3.1.1. Scoring in *MrBUMP*

**Figure 4:**
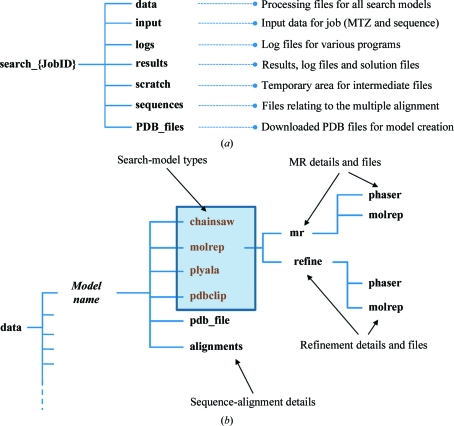
(*a*) The top-level directory structure for a *MrBUMP* job. All job information is stored under a folder ‘search_JobID’, where JobID is the job identifier. The ‘data’ directory contains all of the processing data for each of the template search models. (*b*) The breakdown of the ‘data’ directory. There are potentially four model types created for each template search model. Each model-type directory contains the molecular-replacement and refinement files for that model.

*MrBUMP* is not designed to give a final model for the target structure. Its main objective is to derive the best possible solution to the MR problem from the resources that are made available to it. This model or the corresponding generated electron-density map can be further processed in one of the popular automated model-building programs that are available [*e.g. Buccaneer* (Cowtan, 2006 ▶) or *ARP*/*wARP* (Langer *et al.*, 2008 ▶)] and then by hand in a graphical model-building program (*e.g. Coot*; Emsley & Cowtan, 2004 ▶) to generate the final completed structure. It is only at the model-building stage that it really becomes clear whether the MR solution is a viable one. *MrBUMP* provides a rough guide on how good a solution is through the restrained refinement with *REFMAC* that is carried out on each of the search models that are successfully positioned in MR. The behaviour of the *R*_free_ value during these cycles is monitored and an assessment is made based on the final value and how the value has changed over the course of the refinement. The criteria for the solution-quality assessment are as follows:
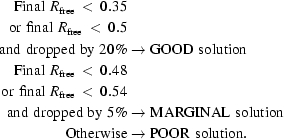
It should be noted that this is a conservative scoring system and that even poor solutions can merit further investigation. In addition to the refinement statistics, users should also look at the scoring from the molecular-replacement programs when assessing the solutions. These are summarized in the *MrBUMP* log file, and the entire MR program log file for each of the search models is stored if further investigation is required (see Fig. 4 ▶).

#### 3.1.2. Program output

At the end of a *MrBUMP* job the log file presents the user with the complete list of processed search models, their solution-quality assessment and the final *R*_free_. The solutions are ranked according to the final *R*_free_ value. The resulting output files for the best solution are also presented at the end of the log file and if the user wishes to access the files for a different solution then these are listed in the log file at the point where the model has been processed.

One of the key benefits of using *MrBUMP* in a molecular-replacement problem is its ability to try many possible models in MR. This can result in the production of a large amount of data and requires the program to have efficient and intuitive cataloguing of this data. Fig. 4 ▶ gives an outline of the file tree that is created by *MrBUMP*. A top-level directory (Fig. 4 ▶ *a*) is created called ‘search_JobID’, where JobID is the job number assigned by the *ccp*4*i* interface if run through *ccp*4*i* or the value assigned to the JOBID keyword variable if run from the command line. Below this, several directories are created. The most important of these is the ‘data’ directory, which stores all of the data-processing information for the template search models. This includes the model-preparation data, MR data and refinement data. Fig. 4 ▶(*b*) shows the ‘data’ directory tree for an individual template search model.

#### 3.1.3. *MrBUMP* and clusters

A useful feature of *MrBUMP* is that it allows the farming out of molecular-replacement jobs to a compute cluster where available. It currently supports the Sun Grid Engine (SGE) and Portable Batch System (PBS) queuing systems. When running from the command line, enabling the ‘CLUSTER’ keyword will cause the program to submit each of the MR jobs to the job queue. In the *ccp*4*i* interface the option to submit jobs to the queue system is presented automatically if either of the systems mentioned above are detected. The master process will then monitor the queue to determine when jobs have completed. This can help to speed up the processing of the template search models and can also allow the testing of a larger set of search models in molecular replacement.

#### 3.1.4. Case study: using *MrBUMP* on a cluster at CSIRO

At Australia’s Commonwealth Scientific and Industrial Research Organization (CSIRO), we have expressed proteins from certain genes taken from some bacteriophages that infect the bacterium *Lactobacillus lactis*. This bacterium is used in the dairy industry to make cheese and buttermilk. The bacterio­phages can cause significant product-quality problems owing to their ability to stop the bacteria converting lactose to lactic acid (Coffey & Ross, 2002 ▶).

While many of the bacteriophage genes express proteins of known function, there are some that are not known. Typically, the proteins are expressed in *Escherichia coli* and then crystallized at the Collaborative Crystallization Centre (C^3^) facility. The resulting crystals are exposed to X-rays at the Australian Synchrotron and a set of diffraction images are collected. These diffraction images are processed into an MTZ file using *MOSFLM* (Leslie, 1999 ▶). The set of residues in the protein is known from the expression process and is formatted into a *FASTA* sequence file.

Once we have the MTZ file and the *FASTA* file, *MrBUMP* can be used to attempt structure solution. In our first attempt, using the built-in *DOFASTA* option, the first few hundred trials did not yield a similar protein or a good molecular-replacement solution. To be certain that there was indeed no suitable protein available, a brute-force search for models was attempted. The versatility of *MrBUMP* and its clustering functionality allowed us to perform a widescale search of structures in the PDB on a compute cluster to determine whether or not there was a suitable model present in the PDB. We used a nonredundant subset of 10 000 of the near-70 000 entries in the PDB. A shell script was used to divide up the set of search models into small groups of about 160 and feed them into *MrBUMP via* the INCLUDE keyword. This is necessary because all processes on the cluster have a specific time limit, so *MrBUMP* is limited to processing about 160 entries at a time. Another useful option to the program is the QSIZE keyword, which we used to limit the number of processes on the cluster queue to equitably share the cluster with other users. Our testing confirmed that there were no suitable models in the PDB. The method developed here shows how using *MrBUMP* it is feasible to set up a large-scale search of the PDB with relative ease. Given access to relatively modest computing resources it is viable to perform such a search in difficult-to-solve cases. About 6–8 months later a similar protein structure did in fact appear and the structure was solved using molecular replacement (data not yet published).

### 3.2. A large-scale test of *MrBUMP*

To illustrate the usefulness of the program, we have carried out a large-scale survey of the results of *MrBUMP* for a set of 552 randomly chosen PDB structures deposited in 2009. The processing was carried out using the depositions in the PDB up to 2009 as the source for search models. One con­straint we have used is that the data are for structures containing a single molecule only. As such we will not discuss the benefits of the multimer search option in *MrBUMP*. A more comprehensive survey will be carried out using all of the 2009 depositions and will be the subject of a future article, but for the purposes of illustrating the advantages of *MrBUMP* here the chosen set will suffice.

In order to gain an in-depth appreciation of how *MrBUMP* performs, in each example we have enabled many of its features and instructed it to try a wide selection of models. Depending on the results of the *FASTA* search, up to 15 whole-chain search models can be generated as well as the potential for several *SCOP*-based domain search models and an ensemble model of the best-scoring individual models for use in *Phaser*. Of the set we have chosen, 342 produced at least a ‘good’ solution, 98 produced at best a ‘marginal’ solution, 66 produced at best a ‘poor’ solution and the remaining 46 had no suitable search models available in the PDB. 312 of the targets had homologues of at least 80% sequence identity, while 92 of the structures that had suitable search models had homologues of sequence identity 40% or less. Originally, 432 of the set were solved by molecular replacement.

The tests were carried out on the compute cluster facilities of the CCP4 Group and the Diamond synchrotron facility in the UK. The CCP4 cluster is made up of eight nodes, each with two 2.0 GHz dual-core AMD processors and 4 GB of RAM. The Diamond system has a total of 77 compute nodes. The bulk of its nodes are 2.5 GHz dual-processor quad-core Intel Xeon chips with 16 GB of RAM. For a single run of *MrBUMP*, it is possible to farm out the processing of each of the search models in MR using a cluster-queuing system (currently, the PBS and SGE queuing systems are supported). This is very useful when running a single *MrBUMP* job; however, given the vast number of jobs carried out in this work we opted not to use this facility in these tests. On the older CCP4 cluster the average run time for a *MrBUMP* job was 72 min. On the more up-to-date Diamond machine the average run time was con­siderably faster at about 39 min. It should be noted that in some cases jobs were found to take as much as 24 h to com­plete (on the CCP4 cluster). As with *BALBES*, the length of time it takes to run *MrBUMP* depends on many factors such as the number of search models tested, the size of the target structure and the sequence identity of the target with its homologues.

#### 3.2.1. Finding the best solution

**Figure 5:**
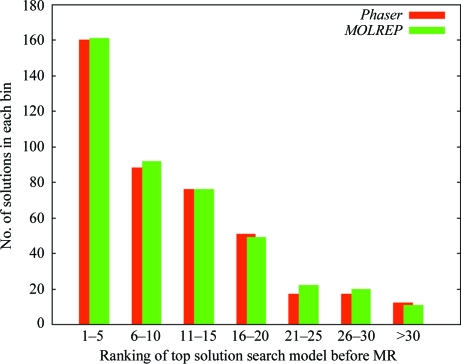
This histogram shows a breakdown of where the top-scoring solution in each of the *MrBUMP* jobs ranked in terms of its initial ranking before being put through molecular replacement. This ranking is based on the score function for the search models. As can be seen, it often happens that the top-scoring solution lies outside the first five top-scoring search models.

When the model search and preparation stage of *MrBUMP* has completed, the list of search models that will go forward to the MR stage are ranked in accordance with a score derived from the alignment of the template sequence with that of the target. Making the choice of best search model based on sequence identity is similar to what a user might do if they were performing a blind search for potential models manually. At the end of a *MrBUMP* job each of the models are again ranked according to how well they refined in *REFMAC*. Fig. 5 ▶ shows the distribution of where what has been deemed the highest ranking solution in each of the *MrBUMP* jobs (post-refinement) lies in terms of how that specific model was ranked before MR was initiated in the pipeline. Only ‘good’ or ‘marginal’ solutions have been considered here. The histogram clearly shows how the best solution quite often lies outside the first five molecules tested and can even be found beyond the 30th ranked search model. *MrBUMP* provides the user with the ability to test a large set of search models and identify the one that is likely to provide the best possible starting point for model building. As demonstrated here, this best solution quite often does not stem from what the user might anticipate as the best search model. Also, in cases where a solution is hard to find the exhaustive nature of *MrBUMP* provides a means of finding that solution.

#### 3.2.2. Using different model types

**Figure 6:**
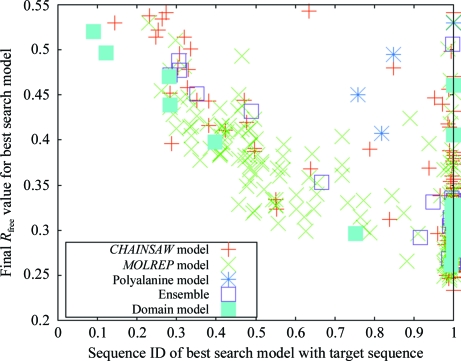
A plot of the model types for the best-scoring search models after molecular replacement and refinement for each of the *MrBUMP* jobs in the test set. We only show results where a solution was deemed to be ‘marginal’ or better.

As outlined above, the model-preparation step in the *MrBUMP* pipeline allows the creation of several different model types for each template search model. In Fig. 6 ▶ we show for each of the *MrBUMP* jobs in the test set what type of model was used for the best solution after all models have been processed in MR. The figure plots the sequence identity of the search model with the target sequence against the final *R*_free_ for each of the best solutions. Again, we only consider solutions that are marginal or better. As is commonly accepted, where the sequence identity is lower than 30% it is difficult to find a solution. There are a few cases below this threshold where marginal solutions are indicated and we will discuss one of these (example 2zzt) in more detail later.

Examination of the breakdown of model types reveals that the ‘mixed model’ generated by *MOLREP* (green crosses) works best on many occasions. However, the mixed models generated by *CHAINSAW* (red crosses) feature more prominently where the sequence identity is low. This can be explained by the use of a multiple sequence alignment to generate the alignments used by *CHAINSAW* to prepare the template search models. *MOLREP* performs its own pairwise alignment of the search-model sequence and the target sequence, making use of some structural information from the template model. At lower sequence identities (<30%) the accuracy of the multiple alignment is better than the pairwise alignment and potentially produces better search models for molecular replacement. In all of the examples in the test set we have used *ClustalW*2 (Chenna *et al.*, 2003 ▶) to perform the multiple-alignment step because of its speed. We have observed that using *PROBCONS* (Do *et al.*, 2005 ▶) or *MAFFT* (Katoh *et al.*, 2005 ▶) can produce a better multiple alignment, particularly in cases where the sequences used in the alignment vary widely in their lengths.

**Figure 7:**
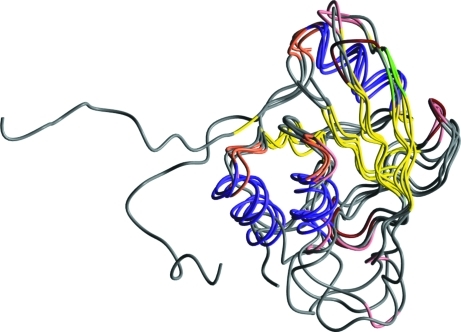
Example 2wfb: crystal structure of the apo form of the orange protein (Apo-Orp) from *Desulfovibrio gigas* (S. Najmudin, C. Bonifacio, A. G. Duarte, I. Moura, J. G. Moura & M. G. Romao, unpublished work). This is an ensemble example where the constituent search models (1rdu_*A*, 1eo1_*A*, 1o13_*A*, 1t3v_*A*) in the ensemble failed to yield an acceptable solution on their own using *Phaser* to perform the MR. The ensemble itself produced a ‘good’ solution. Providing *Phaser* with the ensemble of aligned search models allowed it to downweight the signal from parts of the models which are not conserved across all of the structures, *i.e.* flexible loops and terminal residues. The figure was prepared using *CCP*4*mg* (Potterton *et al.*, 2004 ▶).

Fig. 6 ▶ also reveals that there are several cases where the best solution is produced using an ensemble of the top search models or a domain-based search model. In the cases of targets 2wfb and 3fyq the ensemble of search models has proven to be the only acceptable solution, with the constituent search models proving to be inadequate on their own. Fig. 7 ▶ shows the structural alignment of the constituent search models (chains *A* of 1rdu, 1eo1, 1o13 and 1t3v) used to generate the ensemble search model for 2wfb. The signal from the nonconserved regions of the alignment such as the flexible loops and the N-terminal residues are down-weighted by *Phaser* in molecular replacement. In example 3fyq the individual search models (chain *A* and chain *B* of 3dyj) have a high sequence identity with the target (66%) but they both fail to give an acceptable solution in MR. Again, the signal from the nonconserved regions in their structure alignment is downweighted in the ensemble search model by *Phaser*, allowing a solution to be identified. 3fyq was originally solved using a MAD experiment (Cheung *et al.*, 2009 ▶).

**Figure 8:**
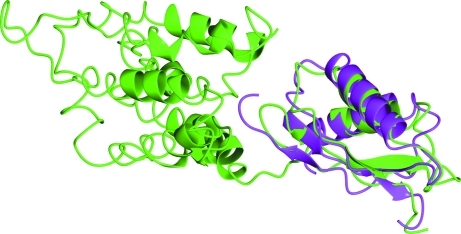
Example 2zzt: crystal structure of the cytosolic domain of the cation-diffusion facilitator family protein. The smaller domain in 2qfi (zinc transporter YiiP; green) matches the structure of 2zzt (pink) very closely in its secondary-structure conformation. The figure was prepared using *CCP*4*mg* (Potterton *et al.*, 2004 ▶).

In Fig. 6 ▶, two example cases where domains were used to give the best solution (2waf and 2zzt) are of particular interest as the search models used had very low sequence identity with the target sequence (<13%). On closer examination 2waf proved to be a negative result, but the 2zzt example solved with a domain-search model with 12.1% sequence identity did prove to be valid and model building could produce a structure for the target. We can see why in Fig. 8 ▶, which shows the alignment of the 2zzt structure (pink) with the parent molecule (chain *A* of 2qfi; green) of the domain that was used to find a solution. It is obvious to the naked eye that the smaller domain in 2qfi matches the structure of 2zzt very closely in its secondary-structure conformation. Given the very low sequence identity, this search model could easily be overlooked. Indeed, the structure of 2zzt was originally determined using a MAD experiment despite the existence of this search model at the time (Higuchi *et al.*, 2009 ▶).

## 4. Discussion

Automation is rapidly becoming an essential part of macromolecular crystallography. From crystallization through to structure deposition, it has enabled the speeding up of the process and made the technique available to a much broader group of potential users. In molecular replacement, software developments such as *Phaser* and *MOLREP* as well as model-preparation programs such as *CHAINSAW* have enabled *BALBES* and *MrBUMP* to fully automate the process. Molecular replacement continues to grow in importance as a method for solving the phase problem in protein X-ray crystallography. Both the surveys that we have carried out here and the more comprehensive surveys carried out earlier by the *BALBES* developers (Long *et al.*, 2008 ▶) indicate that a large percentage (70–80%) of deposited structures in the PDB can potentially be solved using molecular replacement.

The goal of both *BALBES* and *MrBUMP* is to derive the best possible solution for the molecular-replacement problem given the target’s experimental structure-factor amplitudes and amino-acid sequence. Their main objective is to produce a solution that can be taken on to model building and refinement to produce a final structure for the target. Although both programs have the same objective, they differ in the details of their functionality. *BALBES* is highly automated and through its customized internal database it can isolate the best possible models from the PDB for processing. *MrBUMP*, on the other hand, takes a more expansive approach to the problem and allows the processing of a broader set of search models in several different ways and facilitates the tweaking of many of its input parameters by the user. Given that computing power is ubiquitous and inexpensive, it is recommended that users utilize all of the tools that are available to them both in easy-to-solve cases and when confronted with a difficult molecular-replacement problem. This includes manually performing MR using *Phaser* or *MOLREP* as well as automatically in both of these pipelines.

### 4.1. Things to try in difficult cases

(i) In cases where only poor solutions can be found, it is still worthwhile investigating these by hand. The pipelines by their nature do not facilitate access to all of the control parameters for the underlying MR engines *MOLREP* and *Phaser*. Running these programs by hand and tailoring their input to the particular problem may yield a better solution.
(ii) Where the resolution is good enough (better than 1.7 Å) *MrBUMP* provides the option to make use of the *ACORN* program (Jia-xing *et al.*, 2005 ▶) to improve the phases *via* dynamic density modification.
(iii) Check all space groups. *BALBES* facilitates the checking of all space groups related to the input target space group. *MrBUMP* will allow the checking of enantiomorphic space groups.
(iv) When utilizing *BALBES* through its web-service interface it may be combined with the *ARP*/*wARP* web-service facility to produce a model for the target.
(v) *MrBUMP* allows the use of different alignment programs to perform the multiple alignment step of the target sequence and the template sequences. Different and possibly better alignments can result in improved search models. The possibilities are *MAFFT*, *PROBCONS*, *ClustalW* and *T-­Coffee*.
(vi) Use the option to test all generated search models in MR. By default *MrBUMP* will exit if a good solution is found, but allowing it to try all models may reveal several potential solutions. For model building, the better the starting electron-density map and/or model is, the easier it will be to complete. It is worth sifting through the various solutions produced by *MrBUMP* to see which one gives the best starting point.

## Figures and Tables

**(a):** An assembly model of several protein chains: ASSEM__2(2a74A+2a74B+2a74C). No. of models = 1.

Model	Sequence used	Similarity	Residues	Monomers
1	1, 2, 3	0.54	1109	5

**(b):** Search models from one structure: PDB code 1n9w_*A*. No. of models = 4.

Model	Chain ID	Similarity	Residues	Multimer?	Domain?	Monomers
1	*A*	1.0 (ENS)	356	Monomer	No	5
2	*A*	1.0 (ENS)	251	Monomer	Yes	5
3	*A*	1.0 (ENS)	284	Monomer	Yes	5
4	*AB*	1.0 (ENS)	712	Dimer	No	2

**(c):** Search models from different structures: multiple domain models from different PDB entries. No. of models = 4.

Model	PDB code	Similarity	Residues	Multimer?	Domain?	Monomers
1	2i0z_*A*_1	0.234 (ENS)	256	Monomer	Yes	1
2	1s3r_*B*_3	0.218 (ENS)	179	Monomer	Yes	1
3	1w97_*L*_2	0.268 (ENS)	41	Monomer	Yes	1
4	3gbn_*H*_2	0.250	32	Monomer	Yes	1

**Table 2:** The final solution summary A structure is suggested by *BALBES*. Its probability of being a solution is 99.0%.

Model index	as1m1
PDB file	results/refmac_final_result.pdb
MTZ file	Results/refmac_final_result.mtz
*R*_initial_/*R*_final_	0.3730/0.2520
*R*_free:initial_/*R*_free:final_	0.4070/0.3100
*Q* factor	0.842

**Table 3:** Tests on *BALBES*

Starting date	Success rate (%)
1 July	78.9
1 August	86.7
15 August	83.9
1 September	83.9
1 October	80.9
15 October	81.5

**Table 4:** The final solution summary for the structures shown in Fig. 3 ▶ A structure is suggested by *BALBES*. Its probability of being a solution is 99.0%.

Model index	sq2st2m1_sq3st1m1_sq1st4m1
PDB file	results/refmac_final_result.pdb
MTZ file	Results/refmac_final_result.mtz
*R*_initial_/*R*_final_	0.403/0.272
*R*_free:initial_/*R*_free:final_	0.427/0.342
*Q* factor	0.829

**Table 5:** The success rates of low-sequence-similarity (<0.30) cases for the last six round of tests

Starting date	Success rate (%)
1 July	13.5
1 August	14.3
15 August	20.5
1 September	28.6
1 October	14.3
15 October	32.4
